# Determining Fractional Urate Excretion Rates in Hyponatremic Conditions and Improved Methods to Distinguish Cerebral/Renal Salt Wasting From the Syndrome of Inappropriate Secretion of Antidiuretic Hormone

**DOI:** 10.3389/fmed.2018.00319

**Published:** 2018-11-30

**Authors:** John K. Maesaka, Louis J. Imbriano, Nobuyuki Miyawaki

**Affiliations:** Division of Nephrology and Hypertension, Department of Medicine, NYU Winthrop Hospital, Mineola, NY, United States

**Keywords:** hyponatremia, fractional urate excretion, cerebral/renal salt wasting, SIADH, salt balance

## Abstract

Our evaluation of hyponatremic patients is in a state of confusion because the assessment of the volume status of the patient and determinations of urine sodium concentrations (UNa) >30–40 mEq/L have dominated our approach despite documented evidence of many shortcomings. Central to this confusion is our inability to differentiate cerebral/renal salt wasting (C/RSW) from the syndrome of inappropriate secretion of antidiuretic hormone (SIADH), syndromes with diametrically opposing therapeutic goals. The recent proposal to treat most or all hyponatremic patients makes differentiation even more important and reports of C/RSW occurring without cerebral disease leads to a clinically important proposal to change cerebral to renal salt wasting (RSW). Differentiating SIADH from RSW is difficult because of identical clinical parameters that characterize both syndromes. Determination of fractional urate excretion (FEurate) is central to a new algorithm, which has proven to be superior to current methods. We utilized this algorithm and differences in physiologic response to isotonic saline infusions between SIADH and RSW to evaluate hyponatremic patients from the general medical wards of the hospital. In 62 hyponatremic patients, 17 (27%) had SIADH, 19 (31%) had reset osmostat (RO), 24 (38%) had RSW, 1 due to HCTZ and 1 Addison's disease. Interestingly, 21 of 24 with RSW had no evidence of cerebral disease and 10 of 24 with RSW had UNa < 20 mEqL. We conclude that 1. RSW is much more common than is perceived, 2.the term cerebral salt wasting should be changed to RSW 3. RO should be eliminated as a subclass of SIADH, 4. SIADH should be redefined 5. The volume approach is ineffective and 6. There are limitations to determining UNa, plasma renin, aldosterone or atrial/brain natriuretic peptides. We also present data on a natriuretic peptide found in sera of patients with RSW and Alzheimer's disease.

## Introduction

Cerebral salt wasting (CSW) syndrome as first proposed in 1950 has gone through a difficult historical path which may in part be due to the failure of the initial report to prove its existence (1, 2). It has evolved from being a nonexistent entity, confused with the syndrome of inappropriate secretion of antidiuretic hormone (SIADH), considered a misnomer for SIADH, used interchangeably with SIADH, can change from CSW to SIADH in a single patient and finally accepted as a legitimate but rare syndrome (3–7). As previously reviewed, the first report of CSW did not prove renal salt wasting (RSW) to be a legitimate syndrome until Cort reported a decrease in chloride space to document the all-important parameter of being volume depleted in a hyponatremic patient with high urinary sodium concentration (UNa) (2, 8). Differentiating SIADH from RSW as it will be referred to throughout this review has been difficult because of identical clinical parameters that characterize both syndromes, such as hyponatremia, hypouricemia with increased fractional excretion of urate (FEurate), concentrated urine, urine osmolality (Uosm) > plasma osmolality (Posm)], urine sodium concentration (UNa) usually but importantly not always > 30 mEq/L and normal renal, thyroid and adrenal function, Table 1.

**Table 1:** Characteristics of SIADH and RSW.

Both characterized by:
• Association with intracranial diseases
• Hyponatremia
• Concentrated urine
• Urinary [Na] usually > 30 mEq/L
• Normal renal/adrenal/thyroid function
• Non-edematous
• Hypouricemia, Increased FEurate
• Only difference is volume status

*Findings common to both SIADH and RSW. Difference between SIADH and RSW include normal to high ECF (extra-cellular fluid) volume in SIADH and decreased in RSW. Less reliable are; decreased plasma renin and aldosterone levels in SIADH and increased in RSW. UNa values in each syndrome may be deceiving*.

The outmoded volume approach, which categorizes hyponatremic patients according to their volume status, such as being euvolemic, hypovolemic or hypervolemic, has been in existence for over 50 years despite a general agreement that we cannot accurately assess the volume status of nonedematous patients by usual clinical criteria (4, 9, 10). A critical clinically undeterminable difference is the volume status of these patients, being euvolemic or actually to be hypervolemic by multiple determinations of intravascular and extracellular volume (ECV) in SIADH and volume depletion in RSW. Studies that utilized the gold standard radio isotope dilution technique to determine blood volume by ^51^chromium labeled red blood cells (RBC) and/or radio-iodinated serum albumin (RISA) showed 40–94% of patients to be volume depleted as seen in RSW as compared to 12.5–50% being volume expanded as in SIADH in hyponatremic neurosurgical patients of multiple etiologies, suggesting that RSW is more common than SIADH in neurosurgical patients, especially those with subarachnoid hemorrhage (SAH). Some studies also demonstrate RSW to occur in normonatremic patients, Table 2 (11–14). Nevertheless, the dominant view amongst internists is to regard CSW/RSW as a rare condition as compared to neurosurgeons, neurologists and critical care physicians who consider RSW to be more common than is perceived by internists. Differentiating SIADH from RSW is, moreover, more than an intellectual exercise because of diametrically opposite therapeutic goals of water-restricting water-loaded patients with SIADH and administering salt and water to volume depleted patients with RSW. In addition, the awareness that the majority of hyponatremic patients are symptomatic with a fourfold increase in fall rates and bone fractures, creates an urgency to resolve this diagnostic and therapeutic dilemma to improve clinical outcomes (15, 16). This diagnostic and therapeutic dilemma was further clouded by reports of RSW occurring in patients without clinical evidence of cerebral disease, thus eliciting the clinically important proposal to change the term CSW in favor of RSW, because RSW would not be considered unless the patient had cerebral disease (17–19).

**Table 2:** Volume studies in neuro-surgical patients.

		**↓Blood Volume in RSW**	**↑Blood Volume in SIADH**	**Urine Na mEq/L**
HN^11^ hyponatremia		10 (83%)	2	41–203
HN^12^ hyponatremia		8 (89%)	1	–
NN^12^ normonatremia		8 (67%)	4	
HN^13^ hyponatremia		17/18 (94%)		43–210
HN^14^ Hyponatremia	F	9/18 (50%)		–
HN^14^ Hyponatremia	M	1/7 (14%)		

*Volume studies in neurosurgical patients by gold-standard radioisotope dilution methods. Note high prevalence of RSW as compared to SIADH, especially in patients with subarachnoid hemorrhage but also with other neurosurgical diseases and RSW occurring in normonatremic patients. HN, hyponatremia; NN, normonatremia; F, female; M, male. Superscript numbers refer to Nelson et al. (11), Wijdicks et al. (12), Sivakumar et al. (13), and Solomon et al. (14)*.

### Developing a new algorithm for hyponatremic conditions

Evaluation of hyponatremic patients took on a significant turn when reports of hypouricemia with high fractional excretion (FE) of urate coexisted with hyponatremia, which led to the proposal that the coexistence of hyponatremia and hypouricemia, defined as serum urate < 4 mg/dL, differentiated SIADH from most other causes of hyponatremia (20). Others found a similar relationship between SNa, hypouricemia and increased FEurate in SIADH and those with hyponatremia associated with hydroclorothiazide (HCTZ) (21). FEurate was increased to >10% when the patient was hyponatremic but normalized to < 10% when the hyponatremia was corrected by water-restriction (20, 22–26). We encountered 5 patients with hypouricemia and increased FEurate, which persisted after correction of their hyponatremia, suggesting that there was a pathophysiological difference between these patients and those with SIADH (27). The persistently increased FEurate after correction of hyponatremia appeared to be consistent with RSW as was indicated by the insight case with bronchogenic carcinoma who presented with findings consistent with SIADH and RSW, Table 1, but with postural hypotension and reflex tachycardia that were suggestive of ECV depletion. He had a normal CT scan of brain. He experienced severe volume depletion with postural hypotension and dizziness, staggered gait, somnolence and slurred speech while being water-restricted for a diagnosis of SIADH based on the suggestion that the coexistence of hypouricemia and hyponatremia was highly consistent with SIADH (20, 27). His FEurate remained persistently increased after finally correcting his hyponatremia by water restriction and salt supplementation. We were convinced that he had RSW and not SIADH and that the persistent increase in FEurate after correction of hyponatremia might be consistent with RSW and certainly inconsistent with SIADH. Of note is that 3 of the 5 patients in this report had no evidence of cerebral disease, which suggested that RSW can occur without cerebral disease (27). We hesitated to make such a proposal until 25 years later when we had unequivocally proven RSW occurring without cerebral disease, to be discussed below (17, 18).

**Figure 1:**
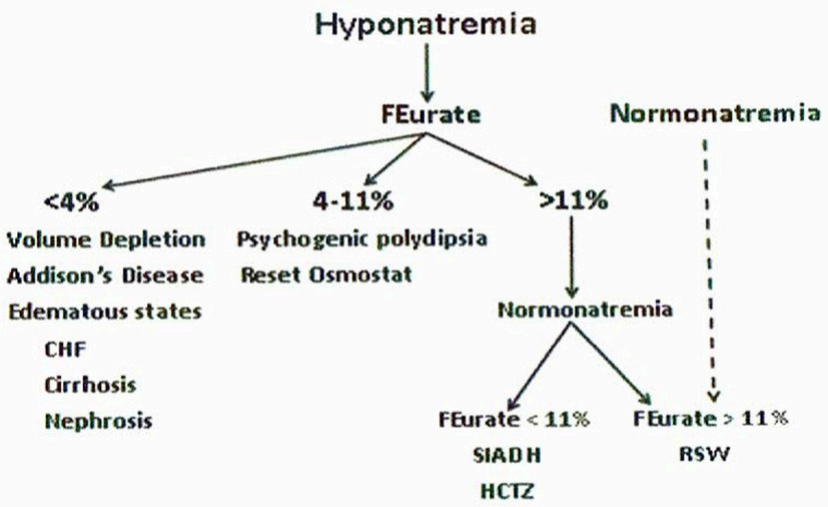
New algorithm based on determinations of fractional excretion of urate in diverse hyponatremic conditions. Assessment of volume status, UNa, plasma renin, aldosterone and atrial/brain natriuretic peptides are not included because of unreliability in the evaluation of hyponatremic patients.

**Figure 2:**
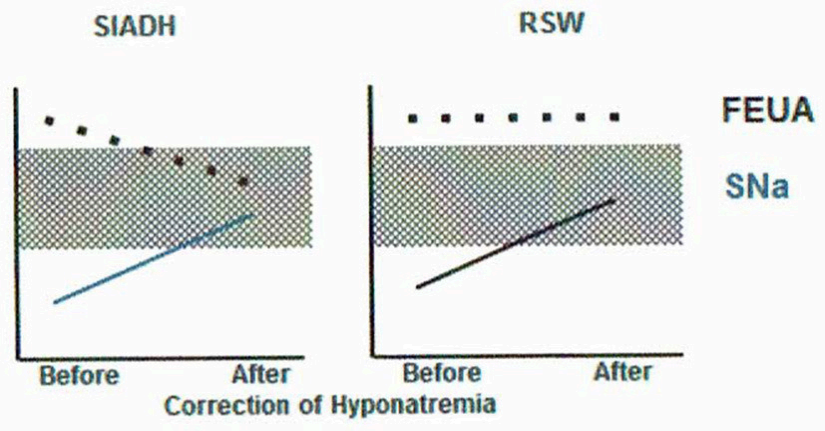
Differentiation of SIADH and RSW based on differences in relationship to serum sodium concentrations and FEurate. There is ample evidence to support this unique relationship between FEurate and serum sodium in SIADH and RSW.

The accumulated experience studying urate metabolism in hyponatremic conditions led to proposal of a new algorithm for evaluating patients with hyponatremia where FEurate reliably identified the cause of hyponatremia in the majority of hyponatremic patients (Figures 1, 2) (2, 10, 17, 18, 27–35). This algorithm eliminates the need to assess the volume status of the patient and minimizes the utility of determining UNa, serum urate, plasma renin, plasma aldosterone and atrial/brain natriuretic peptides. Plasma renin and aldosterone levels have been shown to be affected by too many factors such as angiotensin converting enzyme inhibitors, angiotensin receptor blockers, renin and aldosterone inhibitors, saline infusions, and beta blockers to contribute reliably in the evaluation of hyponatremic patients.

#### Increased FEurate >11%-

We extended our studies of urate metabolism to neurosurgical patients, who were shown to have a high prevalence of ECV depletion and RSW as noted above, and in patients with Alzheimer's disease (AD), who were reported to have hypouricemia (28, 29). FEurate was increased with or without hyponatremia in 29 consecutive neurosurgical patients, in 18 patients with AD as compared to patients with multi-infarct or vascular dementia and age and gender-matched controls and in patients with acquired immunodeficiency syndrome (28–31). Our increasing awareness that normalization of FEurate in SIADH/HCTZ as compared to a persistent increase in RSW after correction hyponatremia was a way to simplify differentiating SIADH from RSW gained significant support from our rat clearance studies. As will be discussed later, injecting the plasma from the same neurosurgical and AD patients with increased FEurate and largely normonatremia into rats revealed the presence of a natriuretic factor that increased FENa and FElithium to provide invaluable data to support our proposal that FEurate helped to identify patients with RSW, including those with normonatremia (29, 36). It would appear that a persistently increased FEurate >11% after correction of hyponatremia appears to identify and potentially differentiate SIADH from RSW. One drawback of this algorithm, however, is the difficult task of correcting the hyponatremia to determine whether the increased FEurate would normalize as in SIADH or remain increased as in RSW (Figures 1, 2). This has been difficult to achieve in a hospital setting, thus limiting the effectiveness of determining FEurate to differentiate RSW from SIADH.

#### Differentiating SIADH from RSW by methods other than FEurate-

As will be discussed in greater detail below, we decided to utilize a reliable under-utilized and unappreciated pathophysiologic difference in the change in urine osmolality in response to isotonic saline infusions between SIADH and RSW to complement studies on the relationship between SNa and FEurate (17, 18, 37). Isotonic saline will eliminate the potent volume stimulus for ADH secretion and permit the coexisting hypo osmolality to inhibit ADH secretion to induce excretion of dilute urines and correct the hyponatremia in RSW but never inhibit ADH secretion or dilute in SIADH.

#### Normal FEurate 4–11%-

We reported a normal FEurate in hyponatremic patients with a reset osmostat (RO) and psychogenic polydipsia (32, 33). We identified 14 consecutive hyponatremic patients with normal FEurate of 4–11% and RO, the diagnosis of which was confirmed by spontaneously excreted dilute urines in 8 or having a normal water-loading test in 6 (32). Differentiating patients with RO from those with psychogenic polydipsia can be simply made by the intake and excretion of large volumes of water and very dilute urines, respectively, in patients with psychogenic polydipsia. A normal FEurate has simplified the diagnosis of RO, which can occur in nearly a third of hyponatremic patients in the hospital (38, 39).

#### Decreased FEurate < 4%-

A low FEurate of < 4% is a common feature of pre renal azotemic conditions where there is increased solute reabsorption in the proximal tubule as seen in edematous states such as congestive heart failure, cirrhosis, nephrosis and extracellular volume depletion with normal renal tubular function. A low FEurate in a hyponatremic patient can also readily identify patients with Addison's disease (34). Because mineralocorticoid deficiency induces renal salt wasting by decreasing sodium transport in the distal tubule, the resulting volume depletion in the presence of normal proximal tubule function increases solute transport in the intact proximal tubule to induce pre renal azotemia that is typified by a low FEurate (40).

Higher FEurates in the 6–8% ranges reported by others in salt depleted patients with hyponatremia suggest that the proposed level of < 4% needs to be addressed in the future, particularly as it relates to medications age and kidney function, (41, 42).

Hyponatremia associated with other conditions such as selective serotonin reuptake inhibitors, amiodarone and myxedema lack sufficient data to incorporate into our algorithm. Losartan and atorvastatin have been reported to increase urate excretion by competing for the anion exchanger in the proximal tubule and increase urate excretion rates, respectively, but we have shown these effects to be minor because a number of our RO patients with normal FEurate were on one or both drugs (32, 43, 44). HCTZ is a well-established cause of hyponatremia and its effect on FEurate is identical to that observed with SIADH by reverting to normal after correction of their hyponatremia while still on HCTZ (21). Implicating HCTZ as the cause of the hyponatremia may be overestimated because we have found persistence of hyponatremia after discontinuing HCTZ (39). Hyponatremia due to HCTZ can be ascertained by stopping the drug and observing dilution of urine and correction of hyponatremia within 2–3 days (45).

### Value of determining fractional excretion of urate

Uric acid is transported exclusively in the proximal tubule by a set of reabsorbing and secretory transporters (46, 47). The determination of FEurate quantitates the net transport of both types of transporters and is expressed as the percent of filtered load of urate that is excreted in urine. It has been shown to be superior to serum urate levels when evaluating patients with hyponatremia and possibly RSW even without hyponatremia as noted above (28–32, 48). Urate transport appears to be controlled by well-defined physiologic phenomena that are in many respects predictable as compared to serum urate levels which are controlled by multiple factors (48). Uric acid is the end-product of purine metabolism with limited degradation by the absence of uricase in humans and humanoids as compared to animals of the lower kingdom. Serum urate levels are determined by variable exogenous and endogenous sources of purine, 30% being excreted through the biliary and gastrointestinal systems and the remaining 70% by the kidneys (48). The complexity of urate formation and excretion has proven to be difficult to arrive at a meaningful definition of hypouricemia, which has been arbitrarily set between 1 and 4 mg/dL (48). We have shown in multiple publications that determinations of FEurate are superior to serum urate to justify our efforts to minimize the value of determining serum urate. We have found FEurate to be increased, normal or low with all combinations of serum urate from low to high in all categories.

#### Saline infusions have a meager effect on FEurate-

There is concern that urate transport is affected by isotonic saline infusions, thus obfuscating the value of determining FEurate under many clinical situations. In a critical review of this subject, saline infusions, whether isotonic, hypotonic or hypertonic have meager effects on urate transport, Table 3 (48–51). For example liberal ECV expansion with isotonic saline robustly increased FENa from a baseline of 1.04–4.43% but increased FEurate only from 7.98 to 9.76%, both of which fall well within the limits of normal (51). FEurate only increased to abnormal levels when FENa was extraordinarily high, Table 3. The following case report exemplifies the failure of saline to affect FEurate.

**Table 3:** Effect of saline infusion on FEurate.

	**FE Na (%)**		**FE Urate (%)**	
	**Cont**	**Exp**	**Cont**	**Exp**
Isotonic	1.04	4.43	7.98	9.76
	1.6	8.2	5.0	5.8
Hypertonic	2.9	18.6	5.4	12.1
	1.4	14.5	12.5	18.7
Hypotonic	1.1	6.1	4.0	7.3

*Studies on the effect of isotonic, hypotonic and hypertonic saline infusions on FEsodium and FEurate in human subjects. Note the very meager effect on FEurate over a wide range of FENa. Cont., control; Exp, experimental*.

### Correcting hyponatremia with hypertonic saline to distinguish SIADH for RSW

**Figure 3:**
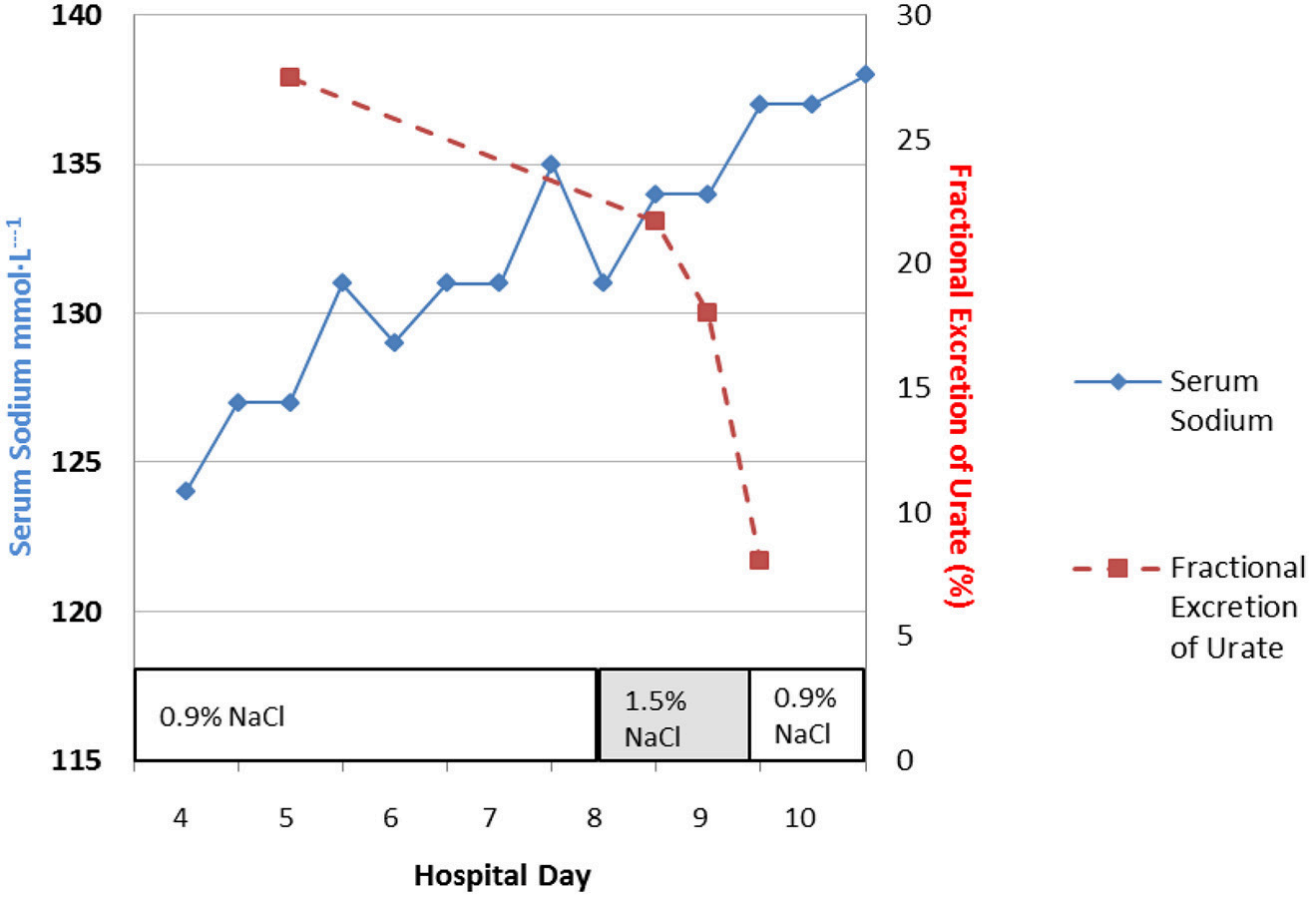
Demonstration of the effect of large volumes of isotonic saline on FEurate in SIADH. Note the gradual normalization of FEurate by correcting hyponatremia after substituting isotonic with hypertonic saline. This graph illustrates the unique relationship between FEurate and serum sodium in SIADH and supports the notion that saline has a meager effect on FEurate. Correction of hyponatremia with hypertonic saline may be one way to differentiate SIADH from RSW as noted in Figure 4.

Perhaps the best example of the failure of ECV expansion to increase FEurate can be appreciated by a patient with a baseline FEurate of 27.5% who had other features that were consistent with SIADH and RSW (Figure 3) (34). The failure of large amounts of isotonic saline to correct his hyponatremia was consistent with SIADH so we decided to correct the hyponatremia with 1.5% hypertonic saline to support our diagnosis of SIADH by demonstrating normalization of FEurate after correction of hyponatremia. As serum Na increased during this process, the FEurate decreased progressively from 27.5 to 8% when his serum Na gradually increased to 138 mEq/L (Figure 3) (34). This very instructive case supports our proposal that ECV expansion with saline has a meager effect on FEurate. This enigmatic relationship between serum Na and FEurate in SIADH remains unexplained. It has been stated that the V1 receptor to vasopressin is responsible for the increase in FEurate in SIADH but DDAVP without V1 receptor activity also reproduced SIADH by inducing hyponatremia, hypouricemia and increased FEurate in normal subjects and equally compelling is the normalization of FEurate after correction of hyponatremia in SIADH despite continued increase in ADH levels with V1 activity (52, 53). Chronic hyponatremia has also been implicated as an explanation for the increased FEurate in SIADH but we have demonstrated normal FEurate in hyponatremic patients with RO, some as long as 10 years (32, 54). On the other hand, the persistent increase in FEurate after correction of hyponatremia in RSW is most likely due to the effect of the natriuretic factor that we demonstrated in neurosurgical patients and in AD (29, 36, 54).

**Figure 4:**
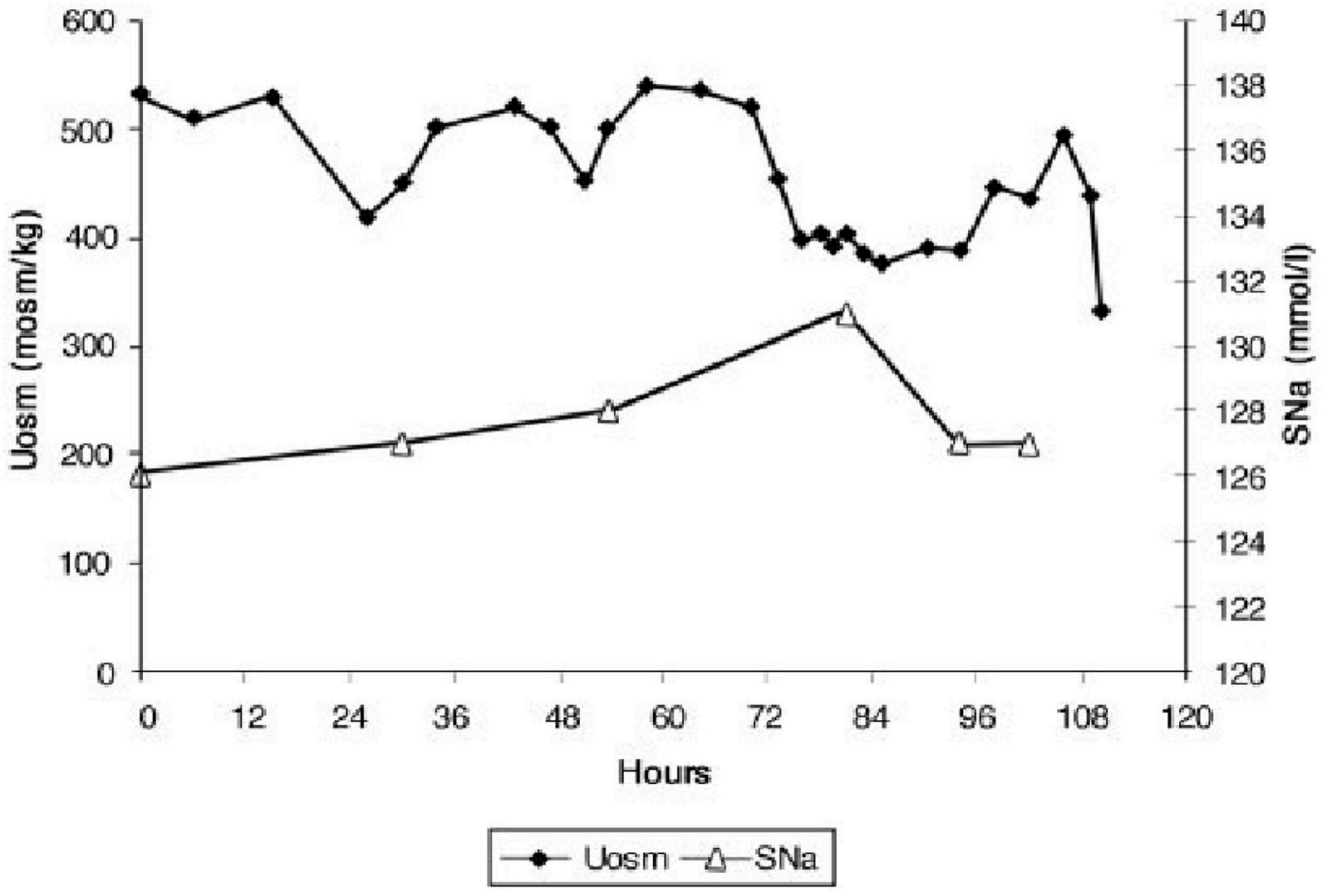
Figure demonstrating the ineffectiveness of isotonic saline in SIADH on urine dilution to comply with the term inappropriate secretion of ADH. Like the patient noted in Figure 7, this patient had RSW without evidence of cerebral disease.

Although FEurate is not increased by intense ECV expansion with saline, it appears that FEurate decreases significantly in volume depleted states as seen in renal Na losses with intact proximal tubular function as in Addison's disease and extrarenal Na losses with intact renal tubular function (34, 40). It is also interesting to note that volume depletion in RSW blunts the increase in FEurate. When ECV is replenished by isotonic saline in the presence of a natriuretic factor, FEurate increases significantly as seen in our hip fracture patient without cerebral disease who had a baseline FEurate of 26.2% which increased to 82% while undergoing volume repletion with saline (Figure 4) (17). It appears that ECV depletion and saline infusions modulate tubular response to a natriuretic factor by decreasing and increasing its effect on urate transport, respectively (17).

### Increased plasma ADH levels are appropriate in RSW and inappropriate in SIADH

#### Isotonic saline infusion in RSW

**Figure 5:**
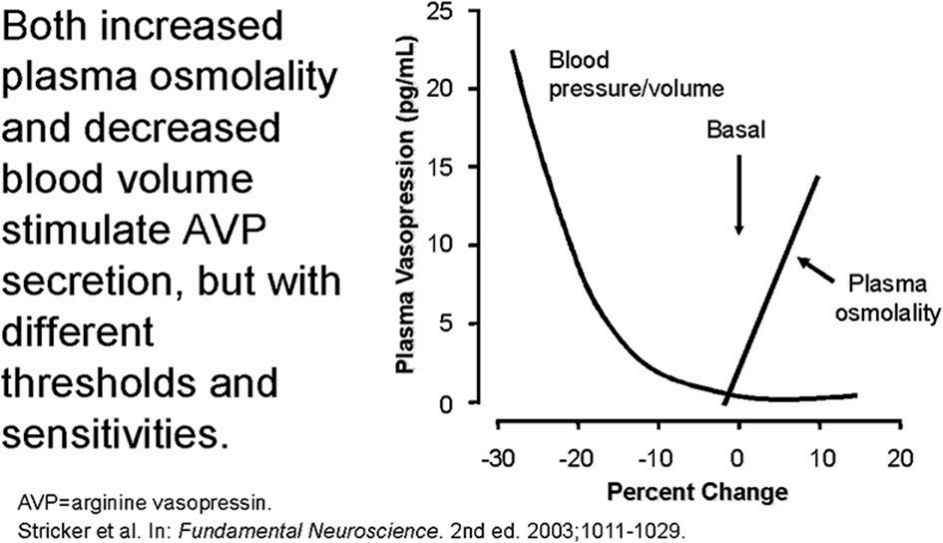
Graph demonstrating more potent effect of volume depletion on ADH secretion as demonstrated in Figure 7. Volume repletion would eliminate the volume stimulus for ADH and permit the hypo osmolality to inhibit ADH secretion.

**Figure 6:**
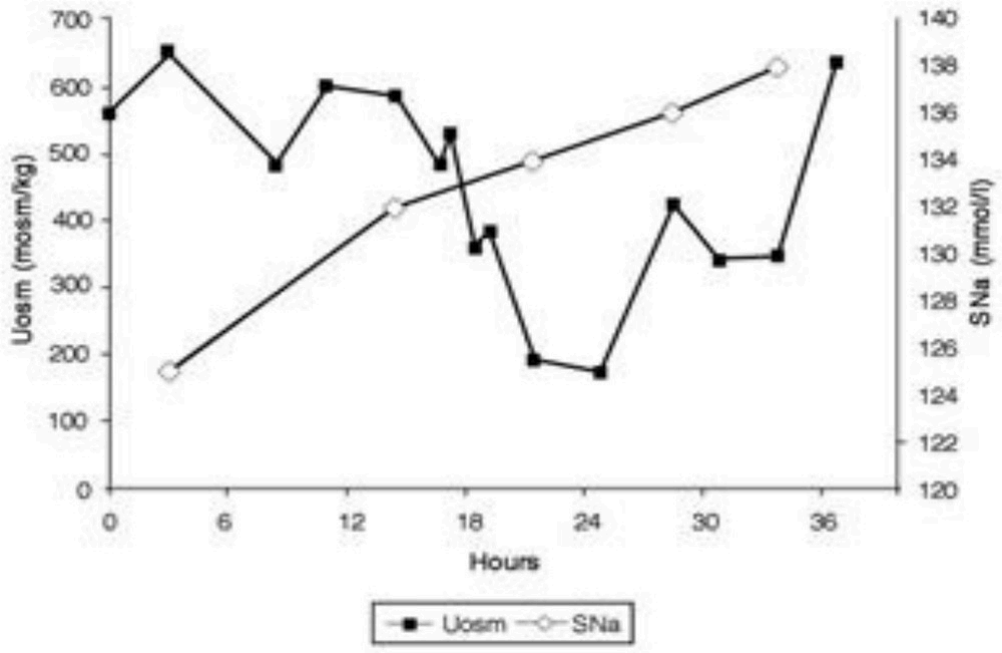
Dilution of urine with isotonic saline in hip fracture patient with RSW without cerebral disease.

**Figure 7:**
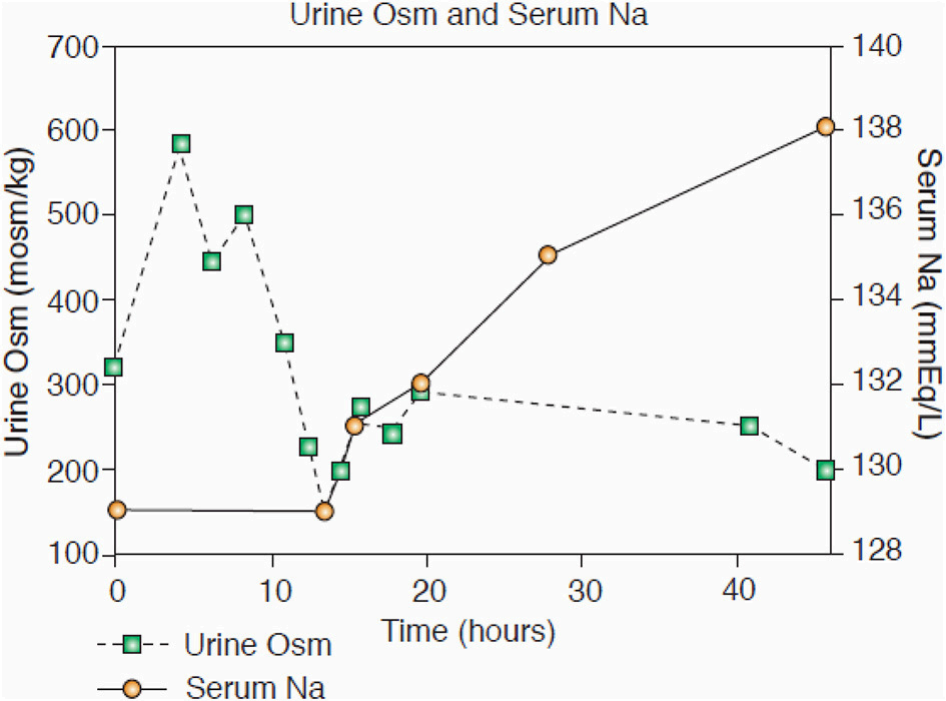
Case of hyponatremic patient with pneumonia without cerebral disease responding to isotonic saline by diluting his urine that is consistent with an appropriate increase in ADH in RSW.

The secretion of ADH in SIADH does not respond to changes in ECV or osmolality of plasma to justify the term inappropriate secretion of ADH (55, 56). This is contrasted to the appropriateness of plasma ADH responses to changes in ECV in RSW. Figure 5 illustrates how the volume stimulus is more potent than the osmolar stimulus for ADH secretion, so that ADH levels remain persistently high while plasma osmolality remains hypo osmolar as long as the patient remains hypovolemic (57). Infusion of isotonic saline to a hypovolemic patient with RSW or extrarenal Na losses with normal renal tubular function will remove the volume stimulus for ADH secretion and permit the coexisting hypo-osmolality to inhibit ADH secretion, thus leading to excretion of dilute urines, increase free water excretion and promptly increase serum Na. Figures 6, 7 illustrate the inhibition of ADH secretion with excretion of dilute urines in RSW as compared to a failure to inhibit ADH secretion with persistent excretion of concentrated urines in SIADH (Figure 4) (17, 18, 57).

#### Isotonic saline infusions in SIADH

In contrast to patients with RSW, there is a common perception that isotonic saline infusions in SIADH fail to inhibit ADH secretion and continue to excrete concentrated urines (Figure 4) (18, 56). In two cases of SIADH in whom radio isotope determinations of blood volume were increased in a patient with neurosurgical disease and another with autonomic dysfunction, isotonic saline failed to induce excretion of dilute urines and correct the hyponatremia (18). These observations are consistent with the view that isotonic saline infusions do not correct the hyponatremia in SIADH and make water restriction a difficult modality of treatment until the advent of the vaptan or V2 ADH receptor inhibitors (56, 58). A review of the literature reveals that dilute urines have been accomplished under specific conditions in SIADH. In the original report of SIADH in 1957, the infusion of isotonic saline at a rate of 16 ml/minute for 2 h decreased Uosm to 170 mosm/kg possibly due to a failure of the dilute urine in the distal tubule to equilibrate with the solute-rich medulla because of the high urine flow rate, possible downregulation of the V2 ADH receptor or intense effect on volume baroreceptors to inhibit ADH secretion (55). Dilute urines were also noted in experimentally-induced SIADH by daily injections of vasopressin in normal human subjects and in dogs while being on a low Na intake of 13 mmol/day or completely eliminating Na from the diet, respectively (59, 60). It would appear that induction of dilute urines with rapid correction of hyponatremia by isotonic saline infusion in RSW and failure to do so in SIADH support our proposal to utilize these distinctly different pathophysiologic responses to isotonic saline to differentiate SIADH from RSW.

#### RSW in hip fracture without cerebral disease

An extremely instructive case is a patient with a hip fracture who was misdiagnosed as SIADH and water restricted for 10 days before being seen by nephrologists. The salient features of this case are outlined below (17).

1. Hip fracture without cerebral disease with hyponatremia and UNa >30mEq/L, misdiagnosed as SIADH by internist. Placed on fluid restriction < 1,000 ml/day for 10 days.
2. When seen by nephrology while on this fluid-restricted regimen, the UNa was 6 mEq/L, FENa 0.11%, Uosm 321 mosm/kg, SNa 129 mEq/L and serum creatinine 0.8 mg/dL.
3. With a UNa of only 6 mEq/L the traditional diagnosis of extrarenal Na losses in the presence of normal kidney function was entertained, but a low serum urate of 3.4 mg/dL was inconsistent with the classic pre renal state where the traditionally low FEurate increases serum urate levels (40). Instead, a markedly increased FEurate of 26.2% was more consistent with RSW/SIADH (Figure 1). The patient was entered into an IRB-approved study.
4. Baseline plasma ADH, renin and aldosterone levels were increased and atrial natriuretic peptide decreased as seen in volume depleted patients.
5. An all-important blood volume study at baseline utilizing gold standard radio isotope dilution methods with ^51^chromium-labeled red blood cells (RBC) and radio-iodinated serum albumin (RISA) revealed blood volume to be decreased by 7.1%.
6. As per protocol, isotonic saline was initiated at 125 ml/h and Uosms determined after every passage of urine. As can be noted in Figure 7, Uosm increased from a baseline of 321 mosm/kg to 587 mosm/kg 4 h after initiating isotonic saline infusion (Figure 6). This increase will be discussed later to support our contention that she was on a low Na diet to yield a UNa of only 6 mEq/L. Uosm proceeded to decrease to a diluted value of 140 mosm/kg when plasma ADH was undetectable to demonstrate the appropriateness of the increase in plasma ADH in RSW. As expected serum Na normalized to 138 mEq/L within 48 hours after initiation of isotonic saline infusion and FEurate remained increased. Plasma renin and aldosterone levels decreased from baseline high levels as would be expected with volume repletion in a volume depleted patient.
7. This case illustrates the appropriateness of the increased plasma ADH levels in RSW where the more potent volume stimulus continues to stimulate ADH production with perpetuation of hyponatremia and when eliminated by volume repletion, permits the coexistent hypo-osmolality of plasma to inhibit ADH secretion (Figures 5, 6). This case documents all of the essential pathophysiological features of RSW, the diagnosis being strengthened by the all-important determination of blood volume by gold standard radio isotope dilution method. It should be noted that the patient woke up 16 h after initiation of isotonic saline infusion saying “I feel so much better, I'm hungry.” Volume depleting a volume depleted salt wasting patient by fluid restriction for an erroneous diagnosis of SIADH increased morbidity by decreasing appetite, decreasing salt intake and excreting a UNa of only 6 mEq/L. Baseline studies before initiating saline infusion included SNa 129 mEq/L, UNa 6 mEq/L, FENa 0.11%, FEurate 26.2%; 19 h after initiation of isotonic saline infusion, SNa increased to 132 mEq/L, FEurate 63%, FENa 1.85%, UNa 90 mEq/L (17). FEurate remained increased after correction of hyponatremia to strengthen our proposal to use this relationship between SNa and FEurate to make the diagnosis of RSW (figure 1, 2).
8. It should be noted that the UNa of 6 mEq/L and FENa of 0.11% would be construed under usual guidelines to be consistent with extrarenal losses with normal renal tubular function but the very high FEurate of 26.2% made it highly unlikely to be part of a pre-renal azotemic state (40). The subtle unusual features extracted from this very instructive case can only be made because it met all of the essential pathophysiologic phenomena that define RSW. The BUN to creatinine ratio did not differ from two cases of SIADH with high FEurate and increased blood volume determinations to eliminate any question of the validity of the diagnosis of SIADH. The inhibition of sodium and water transport in the proximal tubule decreased the necessary urea gradient for urea reabsorption in RSW (17, 18). The reader should spend time to appreciate the wealth of information that can be extracted from this case, many being subtle findings that are considered out of the realm of RSW that will be summarized later.

A similar case showing dilution of urine after initiation of isotonic saline infusion was reported in a patient with pneumonia and absence of cerebral disease (Figure 7) (18). In this case ADH was not determined at the time that the Uosm was dilute but a normal water-loading test after volume repletion proved the diagnosis of RSW.

#### Review of salt balance in diverse conditions: clarification of misconceptions

There appears to be a common misconception that SIADH and RSW are characterized by UNa that exceeds 30 and even 40 mEq/L and that lower values essentially rules out both syndromes (61, 62). This misconception can in part be traced to definitions of both syndromes which state that UNa is typically >30 mEq/L without stressing that it can be lower and is thus construed as always >30 mEq/L. At this time it would be appropriate to discuss salt balance in different situations.

#### Salt balance in normal humans

A normal kidney has an innate sense or establishes a set point of normal extracellular volume and attempts to maintain this volume by various compensatory hemodynamic and neurohumoral mechanisms. The amount of salt required to maintain this set point for ECV is virtually zero as evidenced by studies in Yanomama Indians, the “no salt Society” (63). With sodium requirements being virtually zero, acculturated societies with high salt intake are above their set point of ECV but like the Yanomama Indians, are equilibrated from day to day where Na intake = Na output (63, 64).

#### Extrarenal na losses with normal kidney function

Studies by McCance and Strauss concluded that extrarenal Na losses will decrease ECV to below the set point so Na excretion is extremely low until the Na deficits have been replaced (65, 66). Once the Na losses have been replaced, Na begins to appear in the urine and eventually reach a new steady of Na balance where input = output. The patient could be on a normal Na diet but UNa will remain low as long as the patient is volume depleted, which is part of the picture of prerenal azotemia where the proximal tubule increases solute reabsorption to give rise to the hallmark increase in BUN to creatinine ratio but also decrease FEurate and increase serum urate (40). If UNa is high when the patient is volume depleted in the absence of diuretic therapy, it would thus be consistent with RSW, since a normal kidney in a volume depleted patient will conserve Na until the Na losses have been replaced. Unfortunately we are unable to assess ECV accurately to make this distinction as discussed above.

#### Salt and water balance in SIADH

The term SIADH as first described in 1957 rightfully gained enormous respect of clinicians and basic scientists because it applied data from the experimental induction of SIADH by administration of pitressin to normal human subjects without the ability to determine ADH levels in plasma (55, 67). It is evident from balance studies in experimentally induced SIADH in human and animal models that increasing water intake was an essential feature of the syndrome. Low intake of water failed to induce the cascade of physiologic changes that characterize the syndrome. Daily injections of pitressin with adequate intake of water initially causes water retention with concentrated urines and increased Na excretion to place the subject into negative Na balance. In time however, they enter a phase of “AVP escape” by down-regulating the V2 receptor for vasopressin and go into Na and water balance with lower urine osmolality that are still in the concentrated ranges, Uosm>Posm, but with increased body weight, body water and reduced total body Na (68). UNa can, therefore, be low if Na intake is low at a time when the subject is in an equilibrated state. In this equilibrated and volume expanded state plasma renin and aldosterone are decreased and atrial natriuretic peptide levels increased (17, 18). While in an equilibrated state, however, Na excretion increases whenever there is an acute increase in water intake (53, 54). Excretion of dilute urines are rarely encountered but have been noted when isotonic saline was given at a rate of 16 ml/minute for 120 min as noted above (55). Dilute urines have also been demonstrated in subjects given daily injections of pitressin when human subjects were placed on a low salt diet of 13 mmol/day or no salt diet in dogs (59, 60). Studies of ECV by determinations of chloride and sulfate spaces and blood volume by ^51^chromium labeled RBC and/or RISA have consistently demonstrated an increase in ECV and blood volume in SIADH (11–14, 18, 55). The volume approach which has been in existence for over 50 years is not only incorrect but remains as the dominant approach to evaluating patients with hyponatremia despite our awareness that we cannot accurately and consistently determine the volume status of patients by usual clinical criteria (4, 9).

### ECV depletion in RSW

#### Concept of “natriuretic escape”

In RSW, a diverse group of intercurrent illnesses upregulate a natriuretic factor which inhibits Na and possibly urate transport in the proximal tubule. The status of ECV or body weight will depend on the balance between sodium input and output and degree of Na transport inhibition by the natriuretic factor. Na output does not exceed Na input for any extended period of time because hemodynamic, neurohormonal and possible renal tubular remodeling are activated to prevent total loss of exchangeable Na from the body. The body, thus, escapes the Na losing effect of the natriuretic factor to maintain a new equilibrated state where Na input = Na output but at a lower ECV and body weight. The body thus escapes the salt losing properties of the natriuretic factor without which Na output will continue to exceed Na input to a point where all exchangeable Na will be lost from the body, which does not happen. This escape from the natriuretic factor probably occurs within a few days and the patients are usually in Na balance by the time they are seen. There is a reduction in total body Na but a Na balance study at a time when the patient is in this equilibrated state will not show Na excretion to exceed Na input, see below. This distinction must be made when discussing Na deficits in RSW.

This escape from the salt losing effect of the natriuretic factor can be referred to as “natriuretic escape” which may mimic the complicated physiologic adjustments made in the resistance to diuretic therapy in heart failure (67).

#### Deoxycorticosterone acetate (DOCA) escape

**Figure 8:**
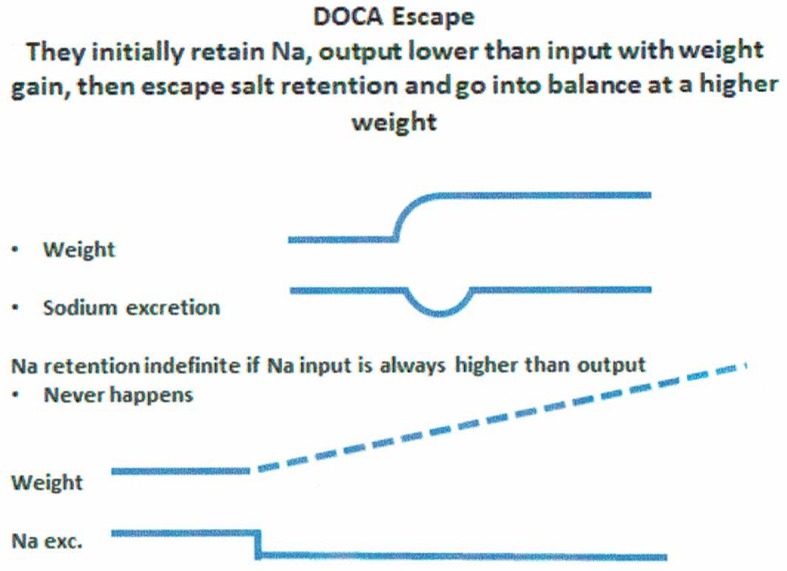
Figure demonstrating the concept of DOCA escape where normal subjects received daily injection of a salt retaining mineralocorticoid, desoxycorticosterone acetate (DOCA), while on a constant salt intake. Sodium excretion was initially lower than intake to increase sodium retention and weight gain only to escape the sodium retaining properties of DOCA to reach an equilibrated state where sodium input matched sodium excretion but at a higher weight from the initial sodium retention. If there was not “escape” from the sodium retaining properties of DOCA, sodium retention would persist indefinitely, which does not happen.

An example of salt balance can be appreciated by understanding the concept of “DOCA escape.” Normal subjects on a fixed Na intake who are given daily injections of DOCA will initially retain Na, Na intake > Na excretion, to increase sodium retention and body weight. However, after essential physiologic adjustments, these patients go into Na balance where Na intake = Na output and weight stabilizes at a level higher than at baseline (Figure 8), despite continued administration of DOCA. This nullification of the Na retaining properties of DOCA is called “DOCA Escape” (70). If the subject never escapes the Na retaining properties of DOCA i.e., Na intake is perpetually >Na output, the subject will continue to retain Na and maintain Na retention as long as Na intake is greater than output, lower half of Figure 8. Instead the subject undergoes “DOCA escape” to re-establish a necessary equilibrated state but at a higher body weight while continuing to receive the same dose of DOCA. DOCA escape is a well-known physiological phenomenon that has provided valuable insights into our understanding of salt balance for over 50 years. At equilibrium, therefore, a low Na diet will result in equally low UNa as can be seen in RSW and SIADH.

#### Approach to urinary sodium concentration (UNa) in hyponatremic conditions

**Figure 9:**
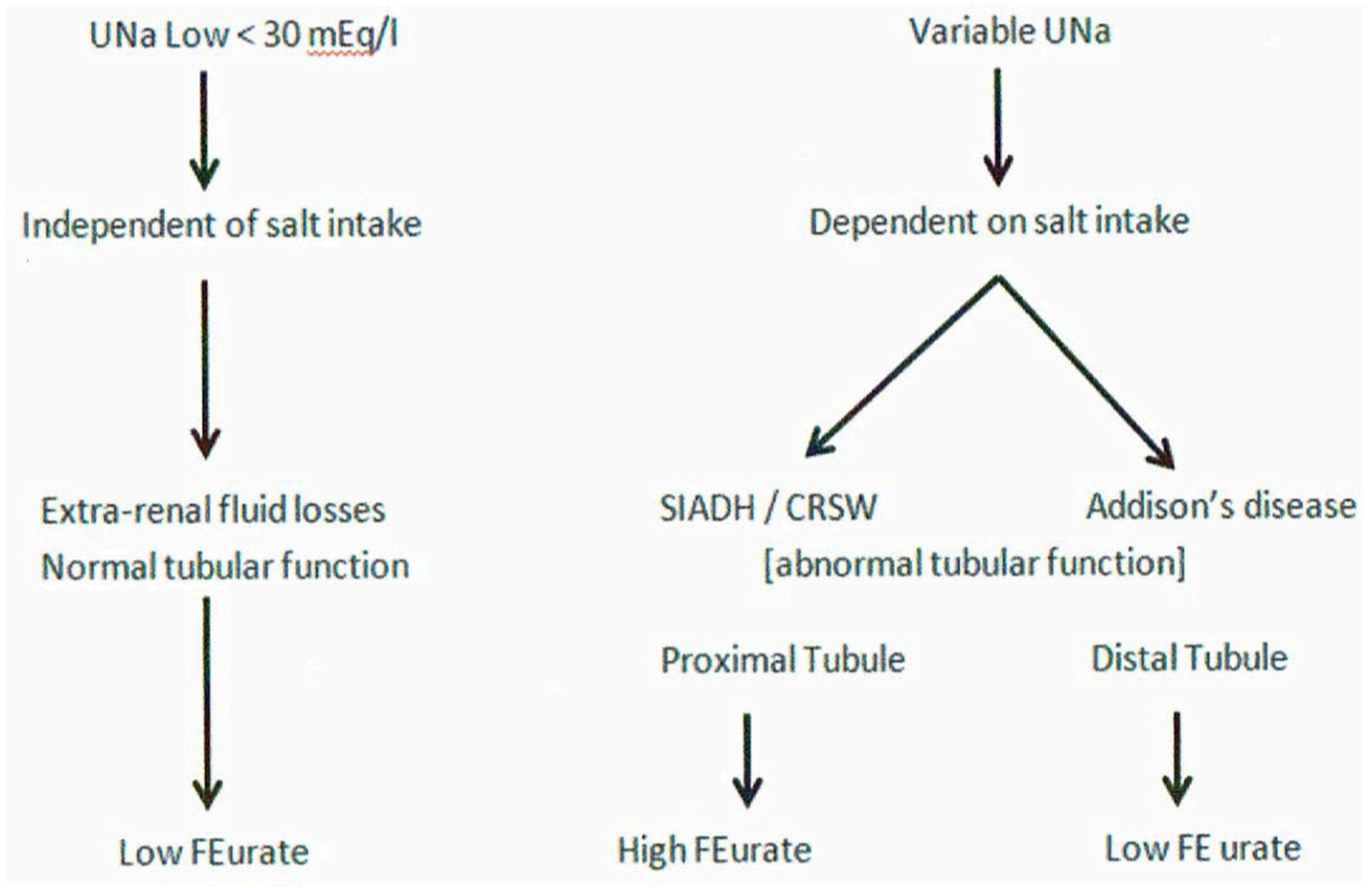
This graph illustrates the superiority of determining FEurate in hyponatremic conditions in which UNa can vary considerably in different clinical conditions in which the etiology can be diverse.

It would appear from the above discussion that UNa has very limited value in assessing patients with hyponatremia although there is a low UNa in pre-renal states that is unrelated to Na intake. UNa, however, reflects Na intake in conditions with defective renal tubular function at different segments of the nephron, such as the proximal tubule in SIADH and RSW and the distal tubule in Addison's disease. A low UNa in a hyponatremic patient can, therefore, be seen in a large number of patients whose hyponatremia can be attributed to many different causes. To this end, we have devised an algorithm where determinations of FEurate can accurately identify whether the hyponatremia is due to extrarenal Na losses with normal tubular function as compared to SIADH/RSW and Addison's disease (Figure 9). A low FEurate will be seen in patients with a low UNa due to extrarenal Na loss and Addison's disease where proximal tubular function is normal as compared to a high FEurate associated with a low UNa in patients with RSW or SIADH. The low UNa in Addison's disease will have a low FEurate because the salt wasting is due to Na losses from the distal nephron with an intact proximal tubule where ECV depletion will induce a prerenal state with increased urate reabsorption, low FEurate and increased serum urate.

#### Further comments on low UNa reflecting low NA input in RSW

It has now been clearly established that a natriuretic factor that predominantly affects Na transport in the proximal tubule can enhance our understanding of RSW. It is very interesting to note that the hip fracture patient without cerebral disease discussed above, had a baseline UNa of only 6 mEq/L with a Uosm of only 321 mosm/kg, which is consistent with a weakened renal medulla resulting from a prolonged low Na intake (Figure 6) (17). The reduced Na concentration in the renal medulla did not provide the proper solute concentration to increase water reabsorption to concentrate the urine despite the presence of ADH. It is remarkable how the first urine excreted only 4 h after initiation of isotonic saline infusion increased Uosm to 587 mosm/kg. As noted in our rat clearance study, the decreased Na transport in the proximal tubule accelerated the increase in sodium delivery to the loop of Henle at the beginning of the outer medulla to increase medullary solute content and increase Uosm. In our view, the UNa of only 6 mEq/L at baseline in a patient with RSW could be made with confidence by demonstrating a decreased blood volume to prove unequivocally the diagnosis of RSW and providing insights into other unusual features of RSW. The low Uosm at baseline reflects a low intake of Na after losing her appetite by being fluid restricted for an erroneous diagnosis of SIADH (17).

#### Lessons from case of RSW in edematous patient with B cell lymphoma

An extremely instructive case was a patient with advanced B cell lymphoma without evidence of cerebral disease, who presented with a 20 lb increase in weight with increasing leg edema of 2 months duration, significant postural hypotension with reflex tachycardia, ascites, pleural effusion and a low cardiac output. He was hyponatremic with SNa of 115 mEq/L, creatinine 0.9 mg/dL, serum urate 6.8 mg/dL, Uosm 308 mosm/kg, UNa of only 10 mEq/L and FEurate 22.7%, which was inconsistent with heart failure (Figure 1). A diagnosis of RSW and obstruction of inferior vena cava was made on admission on the basis of a high FEurate and postural hypotension with reflex tachycardia despite the UNa of only 10 mEq/L, ascites, pleural effusion or decreased cardiac output. He was started on isotonic saline infusion and 10 h later the Uosm was dilute at 140 mosm/kg at which time plasma ADH was undetectable as would be expected in RSW. As discussed in the hip fracture patient without cerebral disease above, the UNa of 10 mEq/L and Uosm of 308 mosm/kg are also consistent with a low salt intake and decreased sodium content of the renal medulla. SNa increased rapidly so 5% dextrose in water was infused to retard the increase in SNa to prevent osmotic demyelination. The diagnosis of congestive heart failure had been made by others based on the pleural effusion, edema, decreased cardiac output and UNa of only 10 mEq/L and he received intravenous furosemide against advice. His urine output increased dramatically to an extent where he had to be transferred to the intensive care unit to administer large volumes saline to establish hemodynamic stability. This very informative case illustrates the failure of the volume approach, the limitation of UNa and the value of FEurate in ferreting out the cause of hyponatremia in this case (34).

#### Treatment of RSW

From our understanding of RSW, increasing salt and water intake is the most appropriate therapy but this approach has been associated with a decreased quality of life by significantly increasing urine output and increasing nocturia to every 2–3 h depending on the potency of the natriuretic factor. In the hospital setting, we infuse isotonic saline at a rate ranging from 75-125 ml/hour for at least 2 days in patients with FEurate > 11% while vigilantly monitoring for any signs of heart failure, especially in the elderly, and changes in the level of serum sodium to avoid too rapid or over correction. Monitoring urine osmolality for at least 36 hours can help to identify patients with RSW if the urine is dilute, Posm > Uosm. On the other hand, infusion of isotonic saline in SIADH would not dilute the urine or correct the hyponatremia (18, 34). There are two conditions in which these patients may not respond to isotonic saline infusions. The first is the phenomenon of desalination where UNa levels remain perpetually increased well above 154 mmol/L so UNa should be monitored carefully to determine whether hypertonic saline infusions should be initiated in this setting to correct or improve the hyponatremia (71). The other possibility is the patient with very high levels of the natriuretic factor where inhibition of renal sodium transport is so severe that isotonic saline cannot overcome the volume depletion associated with it. In this instance, ADH levels are persistently increased because of the potent volume stimulus for ADH secretion and the hyponatremia will be perpetuated, thus requiring hypertonic saline infusions to improve or correct the hyponatremia. In patients with increased FEurate associated with the hyponatremia, it would be helpful to determine FEurate after correction of hyponatremia to at least 137 mEq/L to determine whether FEurate reverts to normal as in SIADH or is persistently increased as in RSW (Figures 1, 2). The amount of salt and water intake in a stable outpatient with RSW can only be estimated because of our inability to ascertain the volume status of the patient. Our approach is to adjust salt and water intake to a point where the patient is clinically comfortable with reduced nocturia. An alternative best solution is to identify and develop an inhibitor to the natriuretic factor (29, 36, 72). Treatment of RSW is further complicated by our inability to determine the duration of RSW.

#### Need to reclassify and redefine SIADH

**Figure 10:**
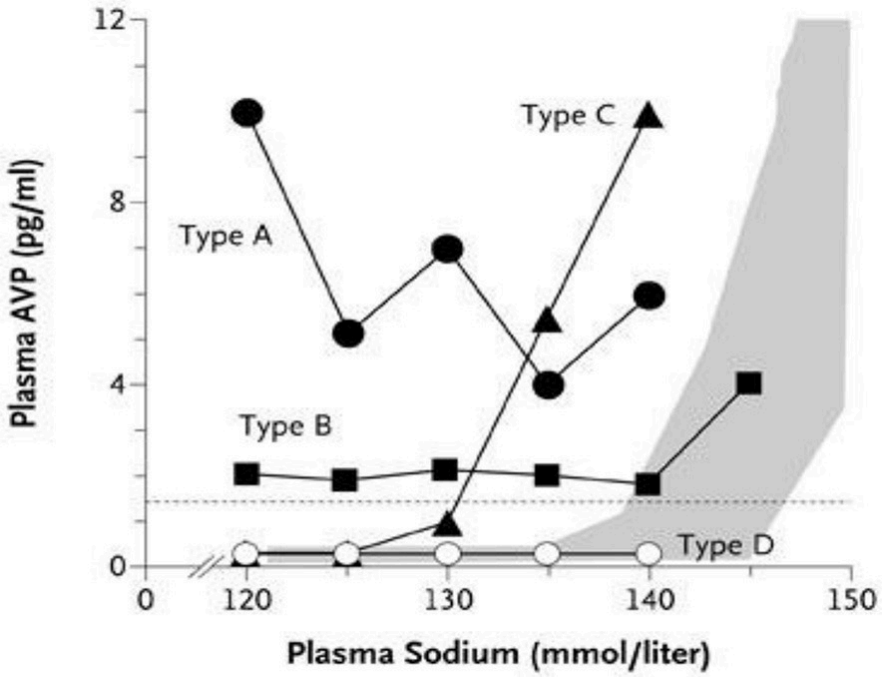
Graph showing the different subclasses of SIADH. Type C is patients with a reset osmostat who have normal FEurates as compared to being increased in the typical form of SIADH. In addition the predictable response to water loading supports our contention that patients with RO represent different pathophysiologic characteristics than SIADH and should thus be eliminated as a subclass of SIADH.

Patients with a reset osmostat (RO), presently classified as type C SIADH, have been shown to have a normal FEurate and predictably normal response to water loading, which are distinctly different from SIADH for unknown reasons (Figure 10) (38, 73). A hyponatremic patient, who is spontaneously excreting a dilute urine, is consistent with RO and psychogenic polydipsia (33, 34). Both conditions are associated with a normal FEurate but differentiating RO from psychogenic polydipsia should be simple by the large volumes of water being ingested by the latter (33, 34). On the other hand. patients with SIADH do not excrete dilute urines under usual clinical conditions. Differences in FEurate and predictably normal response to water loading in RO are pathophysiologically different from SIADH and should therefore be eliminated as a subclass of SIADH (Figure 10) (38, 73). If such is the case, the present definition of SIADH states that the Uosm should be >100 mosm/kg to accommodate RO as a subclass of SIADH (74). We suggest redefining SIADH by eliminating a Uosm of >100 mosm/kg and replacing it with a concentrated urine where Uosm is >Posm.

#### Demonstration of natriuretic activity in neurosurgical and alzheimer's disease patients

It was evident from our early studies that hyponatremic patients with persistently high FEurates after correcting their hyponatremia were pathophysiologically different from SIADH and had clinical evidence of RSW. The critical determinations of blood volume by radio isotope dilution methods demonstrated a large number of neurosurgical patients to have RSW as noted earlier (11–14, 17). These studies prompted us to investigate whether FEurate will be increased in neurosurgical patients where RSW has been reported to be common and to test our hypothesis that a circulating natriuretic factor was responsible for the salt wasting in these patients. The clinical studies demonstrated increased FEurate with or without hyponatremia in a variety of neurosurgical patients (28). At this point it would be helpful to review the characteristics of the natriuretic factor gleaned from our rat clearance studies which demonstrated natriuretic activity in the plasma of neurosurgical patients with high FEurate and mainly normonatremia (consistent with RSW according to our algorithm). Since urate is transported by reabsorbers and secretors exclusively in the proximal tubule (46, 47), we reasoned that a natriuretic factor will most likely affect proximal tubule Na transport but because of the large capacity for Na transport in the distal nephron, we elected to study the transport of lithium, which is known to be transported passively predominantly or exclusively in the proximal tubule on a 1:1 basis with Na in the absence of non reabsorbable solutes such as mannitol (75). The plasma from the neurosurgical patients increased FElithium from 22.3 to 36.6% suggesting that there was a commensurate increase in Na delivery to the distal tubule. It also demonstrated the enormous capacity of the distal tubule to accommodate the increase in distal Na delivery by reabsorbing Na from a control of 23–36.01% as FENa in the final urine increased significantly but modestly from 0.3 to 0.59% (36). These data explain why inhibition of Na transport in the loop of Henle with furosemide in the salt wasting patient with B cell lymphoma had such a profound diuresis (34).

We extended our studies to patients with AD because they were reported to be hypouricemic, which was in part due to an increase in FEurate when compared to patients with multi-infarct or vascular dementia and age and gender-matched normal controls (29). Of the 18 patients with AD only 1 patient was hyponatremic, suggesting a high FEurate with normonatremia would likely identify cases with RSW, Figure 1. As in the study of neurosurgical patients, FElithium increased from a control of 27.2–41.7% with only a modest increase in FENa from 0.33 to 0.63%. Both studies confirmed our proposal that a natriuretic factor in the plasma of these patients had a major effect in the proximal tubule. Blood pressure and GFR were unaffected by the plasma of the neurosurgical and AD patients with natriuretic activity (29, 36). It appears that there is a natriuretic factor that reduced ECV in RSW by inhibiting Na and possibly urate transport in the proximal tubule as compared to patients with ECV depletion due to extrarenal Na losses with intact renal proximal tubule function where there is increased Na, urate and water reabsorption. This difference in proximal tubule function in both conditions explains why the BUN to creatinine ratio was not increased in RSW as compared to SIADH as is proposed by others (17–19). The inhibition of Na and water reabsorption in the proximal tubule in RSW will not increase intratubular urea concentration to create a favorable transtubular urea gradient for passive reabsorption to occur.

We surmised that the natriuretic factor was a small peptide that would be filtered and excreted in urine so we purified ammonium sulfate-precipitated proteins in urine of normonatremic neurosurgical patients of different etiologies and studied transport of ^22^Na across LLC-PK1 cells, a pig proximal tubule cell line, grown to confluence in transwells. We found inhibition of transcellular transport of ^22^Na in a dose-dependent manner in those with high FEurate but not in those with a normal FEurate (70). This study supports our contention that a high FEurate in the presence of normonatremia without a need to go through a phase of hyponatremia is consistent with RSW. We have not tested the plasma of patients with SIADH for natriuretic activity. Atrial/brain, especially brain, natriuretic peptides, which have been implicated as the cause of RSW either directly or by inhibiting aldosterone secretion, are unlikely causes of RSW because of physiologic characteristics that are very different from those observed in the present studies (76, 77).

#### How to differentiate SIADH from RSW

Differentiating SIADH from RSW can be a daunting task because of the perception that RSW is a rare entity, similarities in usual clinical parameters for both conditions and our inability to assess the volume status of patients by usual clinical criteria, Table 1. We have now accumulated enough data to provide information on the best approach to differentiating SIADH from RSW. We utilize two algorithms where FEurate is central to determining the cause of hyponatremia and those with UNa < 30–40 mEq/L (Figures 1, 2, 9). FEurate can distinguish SIADH from RSW by being increased while hyponatremic in both conditions but normalizes in SIADH and remains increased in RSW after correction of hyponatremia (Figures 2, 3). Although there are some data to suggest that an increased FEurate with normonatremia is diagnostic of RSW without going through a phase of hyponatremia, future studies need to confirm this relationship. Moreover, correcting the hyponatremia to see whether FEurate normalizes or remains increased can be difficult to achieve but would be worth attempting because of important diametrically opposite therapeutic goals for both conditions that can significantly affect clinical outcomes. Correction of hyponatremia might be achieved by hypertonic saline infusions to see if FEurate normalizes or remains increased as noted in Figure 2.

An adjunct to determining FEurate is the infusion of isotonic saline to differentiate SIADH from RSW. In RSW Uosm usually becomes dilute, Uosm < Posm, within 24–36 h after initiation of isotonic saline infusion with prompt increase in serum Na, being mindful to monitor serum Na to prevent over correction of hyponatremia to prevent cerebral demyelination (78). By contrast isotonic saline infusions will not induce excretion of dilute urines or correct the hyponatremia in SIADH (18, 57). Administering isotonic saline to an already volume expanded patient with SIADH should be monitored carefully, although these patients are known to rapidly excrete the infused sodium to minimize the possibility of inducing heart failure (55). There should also be concern for inducing desalination by isotonic saline in SIADH and RSW, especially in those with high urinary sodium and potassium concentrations (69). Plasma renin and aldosterone levels will be increased in RSW and decreased in SIADH but can be unreliable because of many interfering circumstances to be reliable as are UNa and BUN to creatinine ratio (17, 18). Serum creatinine should be in the normal range and thyroid and adrenal function should be tested.

#### Evaluation of hyponatremic patients from general medical wards of hospital

We just completed a broad study of hyponatremic patients from the general wards of the hospital outside of intensive care units (39). We utilized our algorithm where determinations of FEurate were central to our approach without any concern for the volume status of the patient with minimal reliance on serum urate levels, UNa, plasma renin, aldosterone or atrial/brain natriuretic peptide levels. We had little or no difficulty identifying patients with reset osmostat, Addison's disease or HCTZ by this algorithm except for differentiating SIADH from RSW because it required correction of hyponatremia to see if FEurate normalizes as in SIADH or remains increased as in RSW (Figures 1, 2). Correcting the hyponatremia, however, can be difficult to achieve in the hospital. Although the infusion of isotonic saline was not part of our research protocol, we took advantage of the frequency with which the clinical staff of the hospital administered isotonic saline to these hyponatremic patients. Isotonic saline eliminates the volume stimulus for ADH secretion to permit the coexisting hypo osmolality to inhibit ADH secretion usually within 36–48 h after initiation of saline infusions in patients with RSW. Excretion of dilute urines to increase free water excretion would then increase SNa, being mindful that an increase in serum of >5 mEq/L while undergoing isotonic saline infusions was most consistent with hypovolemic hyponatremia or RSW (9, 39). By contrast there is the well-known absence of urinary dilution, >5 mEq/L increase in SNa or correction of hyponatremia by isotonic saline infusions in patients with SIADH, as noted above (Figure 4) (18).

Of 62 hyponatremic patients, (a) 27% (17) had SIADH, All of the 17 patients had increased FEurate at a time when they were hyponatremic and all 5 patients who corrected their hyponatremia, normalized a previously high FEurate, all 11 of the 17 patients who received isotonic saline failed to dilute their urine or correct their hyponatremia, and 5 normalized a previously high FEurate after correction of hyponatremia, (b) 31% (19) had a reset osmostat based on normal FEurates in all and spontaneously excreted dilute urines in 8; (c) 38% (24) had RSW, All of the 24 patients had increased FEurate when hyponatremic and 11 patients who corrected their hyponatremia had persistently increased FEurate, 19 of the 24 patients who received isotonic saline, diluted their urine, 2 of whom had undetectable plasma ADH levels when urine was dilute; 10 required 5% dextrose in water to avoid over correction of hyponatremia to prevent cerebral demyelination; 21 had no clinical evidence of cerebral disease, (d) 1 patient had Addison's disease with a low FEurate and (e) 1 due to hydrochlorothiazide.

It is interesting to note that 13 had baseline UNa < 30 mEq/L in RSW, 6 with RO but 4 when urine was dilute and only 3 with SIADH. Since Na excretion reflects Na intake in these conditions, the patients with RSW had more serious comorbid conditions that decreased appetite and Na intake (39). The low UNa in patients that are reputed to have high UNa supports our proposal to minimize the value of determining UNa in hyponatremic patients. Figures 1, 2, 9 illustrate how FEurate can sort out some of the difficulties encountered when UNa is < 30 mEq/L.

An unexpected finding in this study was the large number of patients being identified as renal salt wasters from a population of patients that are not known to have RSW. We feel that our collective method of identification has excellent supportive data to arrive at such a conclusion. The fact that 21 of the 24 patients had no clinical evidence of cerebral disease also strongly supports our contention that the term CSW is certainly an outmoded and a potentially detrimental one in favor of RSW because the diagnosis of RSW would not be considered in the absence of cerebral disease (17–19, 39). This has extremely important clinical implications because these volume depleted patients are being fluid restricted for an erroneous diagnosis of SIADH. There have been cases of increased morbidity of fluid-restricted patients with RSW but this report should alert all physicians to regard RSW as a common entity that should be treated appropriately with salt and water supplementation (17, 27, 79, 80). Until future studies confirm previous perceptions of the rarity of RSW, RSW should be considered a common disorder in the general wards of the hospital. The concern for RSW should remain as a potentially more common disease but an additional challenge is to identify the large number of patients who may have RSW without being hyponatremic and to determine whether a high FEurate without hyponatremia will eventually identify patients with RSW as suggested in our algorithm, Figure 1.

## Summary and conclusions

It is our hope that this review has shed some light into the complexity of evaluating patients with hyponatremia. This complexity can be appreciated by many subtle variations that can be explained by application of basic physiologic principles. This approach will hopefully remove the mystery that would otherwise remain unresolved and lead to inappropriate diagnoses and treatment of a highly diverse group of diseases that make up hyponatremia and related conditions, such as the untapped conditions of RSW occurring without hyponatremia. The reader should focus on the patients with hip fracture and B cell lymphoma who had no evidence of cerebral disease as discussed above to appreciate the subtle messages that bring such clarity to our understanding of many unusual features that were exposed and supported by the certainty of the diagnosis of RSW (18, 34, 39).

We hope we have adequately reviewed the development of a new algorithm that has proven to be effective in identifying many of the causes of hyponatremia without much concern for the determination of the volume status of the patient, presence of edema, levels of serum urate, UNa or plasma renin, aldosterone or ANP/BNP, Figure 1. The complexity of differentiating SIADH from RSW has hopefully been clarified by a better understanding of the unique relationship between FEurate and SNa and the distinct difference in ADH responses to saline to add a well-established physiologic phenomenon to this difficult differentiation. This improved method of differentiating SIADH from RSW led to the unexpected identification of a large number of hyponatremic patients with RSW in the general wards of the hospital. The fact that 21 of the 24 patients with RSW had no clinical evidence of cerebral disease supports our proposal to eliminate the term CSW in favor of RSW because RSW would not be considered if there is no evidence of cerebral disease. This improved method of differentiation will hopefully improve outcomes of two syndromes with diametrically opposed therapeutic goals.

The normal FEurate and predictable ADH response to water loading in RO should eliminate RO as type C SIADH and as a result lead to an improved definition of SIADH by replacing a Uosm of >100 mosm/kg with a concentrated urine where Uosm is greater than Posm. We also hope that we have covered many subtle and important features of salt balance, the concept of being in an equilibrated state under diverse conditions and the variability of UNa even in conditions that are traditionally thought to exceed 30 mEq/L. Finally, the unexpected results of this novel approach to hyponatremia will hopefully stir others to approach hyponatremic conditions with a different mindset and improve or develop better methods than those proposed in this manuscript.

## Author contributions

JM contributed conception and design of the manuscript review and wrote the first draft. LI and NM wrote sections of the manuscript and contributed to design. All authors contributed to manuscript revision, read and approved the submitted version.

### Conflict of interest statement

The authors declare that the research was conducted in the absence of any commercial or financial relationships that could be construed as a potential conflict of interest.
